# A simplified method to correct saturation of arterial input function for cardiac magnetic resonance first-pass perfusion imaging: validation with simultaneously acquired PET

**DOI:** 10.1186/s12968-023-00945-w

**Published:** 2023-06-22

**Authors:** Ran Li, Masoud Edalati, David Muccigrosso, Jeffrey M. C. Lau, Richard Laforest, Pamela K. Woodard, Jie Zheng

**Affiliations:** 1 Mallinckrodt Institute of Radiology, Washington University School of Medicine, 4525 Scott Ave, Room 3114, St. Louis, MO USA; 2 Department of Cardiology, National Heart Centre Singapore, Singapore, Singapore

**Keywords:** Cardiac magnetic resonance, Myocardial blood flow, Arterial input function, Vasodilation, Myocardial flow reserve

## Abstract

**Background:**

First-pass perfusion imaging in magnetic resonance imaging (MRI) is an established method to measure myocardial blood flow (MBF). An obstacle for accurate quantification of MBF is the saturation of blood pool signal intensity used for arterial input function (AIF). The objective of this project was to validate a new simplified method for AIF estimation obtained from single-bolus and single sequence perfusion measurements. The reference MBF was measured simultaneously on ^13^N-ammonia positron emission tomography (PET).

**Methods:**

Sixteen patients with clinically confirmed myocardial ischemia were imaged in a clinical whole-body PET-MRI system. PET perfusion imaging was performed in a 10-min acquisition after the injection of 10 mCi of ^13^N-ammonia. The MRI perfusion acquisition started simultaneously with the start of the PET acquisition after the injection of a 0.075 mmol/kg gadolinium contrast agent. Cardiac stress imaging was initiated after the administration of regadenoson 20 s prior to PET-MRI scanning. The saturation part of the MRI AIF data was modeled as a gamma variate curve, which was then estimated for a true AIF by minimizing a cost function according to various boundary conditions. A standard AHA 16-segment model was used for comparative analysis of absolute MBF from PET and MRI.

**Results:**

Overall, there were 256 segments in 16 patients, mean resting perfusion for PET was 1.06 ± 0.34 ml/min/g and 1.04 ± 0.30 ml/min/g for MRI (*P* = 0.05), whereas mean stress perfusion for PET was 2.00 ± 0.74 ml/min/g and 2.12 ± 0.76 ml/min/g for MRI (*P* < 0.01). Linear regression analysis in MBF revealed strong correlation (*r* = 0.91, slope = 0.96, *P* < 0.001) between PET and MRI. Myocardial perfusion reserve, calculated from the ratio of stress MBF over resting MBF, also showed a strong correlation between MRI and PET measurements (*r* = 0.82, slope = 0.81, *P* < 0.001).

**Conclusion:**

The results demonstrated the feasibility of the simplified AIF estimation method for the accurate quantification of MBF by MRI with single sequence and single contrast injection. The MRI MBF correlated strongly with PET MBF obtained simultaneously. This post-processing technique will allow easy transformation of clinical perfusion imaging data into quantitative information.

## Introduction

Myocardial perfusion, or myocardial blood flow (MBF) is an important index for the diagnosis of ischemic heart disease [1, 2]. Quantitative MBF is not only a measure for functional severity of coronary artery stenosis, but also a pathophysiological indicator of microvascular alterations, which may provide early-stage information about cardiac hemodynamic function. Administration of a gadolinium-based contrast media in cardiovascular magnetic resonance (CMR) through its first passage has become a clinical standard approach to assess myocardial perfusion states [3, 4]. Through model or model-independent constrained deconvolution by using time-intensity or contrast concentration curves obtained from the left ventricle (LV) tissue and blood pool, MBF can be quantified in absolute terms [4]. This approach has been well validated and has been shown to be a robust tool for clinical evaluation of myocardial perfusion and perfusion reserve without irradiation [5–8].

In this quantification process, the myocardial arterial input function (AIF) is usually obtained from the time-intensity curve of a region-of-interest (ROI) placed in the center of LV blood pool area. Because the linear relationship between the signal intensity and the concentration of contrast agent holds for a limited dose of the injected gadolinium agent, the peak signal intensity of AIF may be saturated if the dose of the agent exceeds that limit while the signal-to-noise ratio in myocardial tissue is improved. To overcome this saturation effect on AIF, a dual-bolus approach has been widely adopted [5, 9, 10]. However, there are challenges to performing the dual-bolus protocol during pharmacologically induced hyperemia (adenosine, dobutamine, etc.) when the time available for scanning is limited. For this reason, developing a post-processing method to de-saturate the AIF curve after the administration of a large bolus dose of the contrast agent is an attractive alternative for clinical studies without the added complexity of two injections and two breath-holds with the potential to simplify clinical first-pass myocardial perfusion examinations.

In this project, a post-processing method is introduced to correct the saturated AIF curve for robust quantification of myocardial perfusion. To validate this approach, myocardial CMR perfusion imaging data sets were obtained for a clinical myocardial perfusion research examination to assess for myocardial ischemia performed on a PET-MRI scanner. The ^13^N-ammonia PET perfusion results were used as the reference perfusion values.

## Materials and methods

### AIF interpolation

Based on a single–compartment kinetic model for AIF estimation for liver perfusion quantification, in which there were three concentration terms from two input (artery and portal vein) [11], the myocardial perfusion kinetic model is expressed as (no portal vein contribution) [12]:

1 $$\frac{d{C}_{myo}}{dt}={k}_{art}{C}_{art}\left(t-{t}_{a}\right)-{k}_{myo}{C}_{myo}(t)$$

where *C*_*myo*_ is the myocardial tissue concentration, *C*_*art*_ is the arterial concentration measured in the left ventricular blood pool, *t*_*a*_ is the transit time for the arterial blood to travel to the myocardium, and *k*_*myo*_ and *k*_*art*_ are the respective transfer constants.

**Fig. 1:**
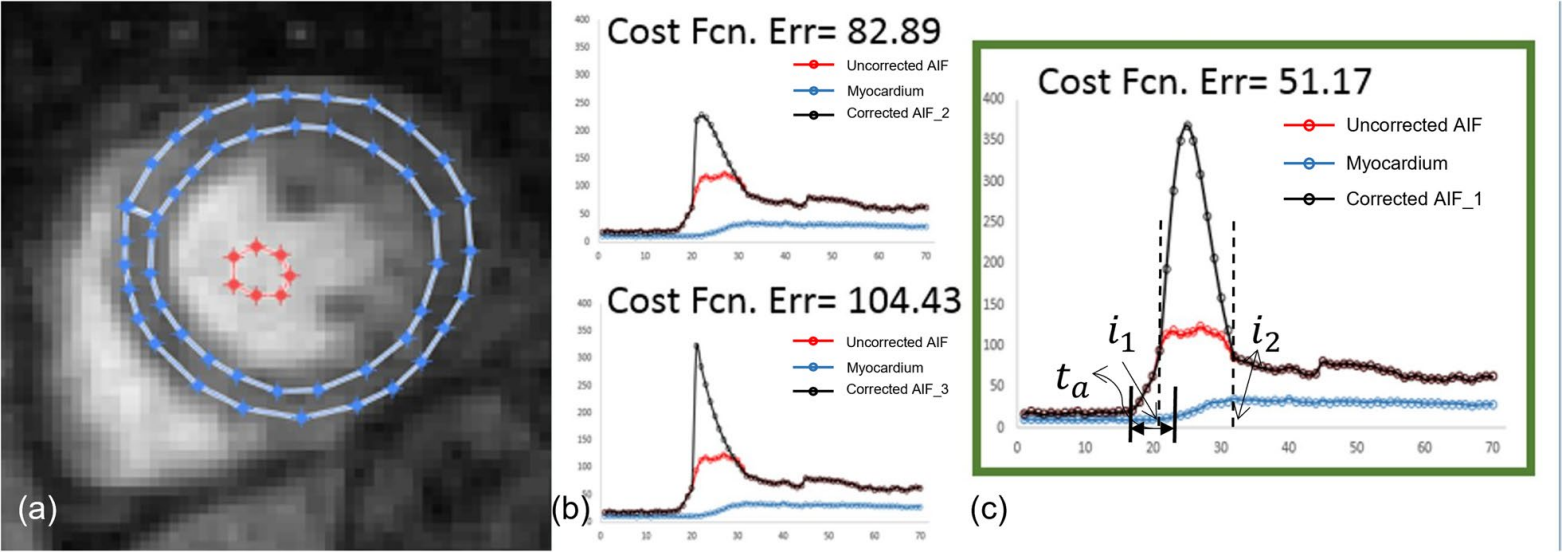
Schematic illustration of the method of AIF estimation. Tissue (blue) and saturated arterial concentration (red) curves were obtained from drawn contours in LV myocardium and blood pool (**a**). Several AIF curves are estimated based on the selection of saturation levels (**b**). A best fit with the lowest cost function error was then determined (**c**). ${i}_{1}$= start time point of the AIF saturation; ${i}_{2}$ = end time point of the AIF saturation; ${t}_{a}$= delay time between the AIF and the myocardial enhancement

Because an AIF is composed of saturated and non-saturated portions, the goal of AIF interpolation is to use the information in the non-saturated portions of the AIF and the entire myocardial concentration curve (non-saturated) to reconstruct a reasonable estimate of the saturated AIF portion. Unlike the spline interpolation approach [8], we opted for a gamma variate model for the estimation of true AIF [9], with a single peak (the saturated portion) between two boundary time points, which we called *i*_*1*_ and *i*_*2*_ (Fig. 1):

2 $$\overline{{C }_{a}}\left(t\right)= \left\{\begin{array}{ll}{C}_{a} & 0\le t<{i}_{1}\\ {m}_{0}\sqrt{\frac{{m}_{1}^{3}}{2{m}_{2}\pi {t}^{3}}}{e}^{\left[-\frac{{m}_{1}}{2{m}_{2}t}{\left(t-{m}_{1}\right)}^{2}\right]}+ b &{i}_{1}\le t\le {i}_{2}\\ {C}_{a}& {i}_{2}<t\le N\end{array}\right.$$

where *b* is the baseline mean, and ${m}_{0}$, ${m}_{1}$, and ${m}_{2}$ are fitting parameters. It was experimentally determined that most original AIFs contained saturated points between 40% (heavily saturated) and 90% (slightly saturated) of the saturated peak. The remainder of the reconstruction was performed according to the previous approach [11], where we built a matrix S = [$\overline{{C }_{a}} {C}_{myo}$] of the curves in vertical vector form, which was used to calculate a cost function E given by:

3 $$E={\left|\frac{\partial {C}_{Myo}}{\partial t}-S\times K\right|}^{2}$$

where

4 $$S=\left({C}_{AIF}\left(t\right), {C}_{Myo}\left(t\right)\right)$$

5 $$K={({S}^{T}S)}^{-1} {S}^{T} \frac{\partial {C}_{Myo}}{\partial t}$$

To determine the corrected ${C}_{AIF}\left(t\right)$, the cost function needs to be minimized under the following boundary constraints:

$${C}_{a}{({i}_{1}-1)}^{{\prime}}\le \overline{{C }_{a}}\left({i}_{1}\right)^{\prime}+\overline{{C }_{a}}{\left({i}_{1}\right)}^{{\prime\prime} }$$

$${C}_{a}{\left({i}_{2}+1\right)}^{{\prime}}\le \overline{{C }_{a}}{\left({i}_{2}\right)}^{{\prime}}+\overline{{C }_{a}}{\left({i}_{2}\right)}{^{\prime\prime} }$$

6 $${C}_{a}\left({i}_{1}\right)\le \overline{{C }_{a}}\left({i}_{1}\right); {C}_{a}\left({i}_{2}\right)\le \overline{{C }_{a}}\left({i}_{2}\right)$$

These constraints enforce the assumptions of continuity at both the border points and in their derivatives. These processes of reconstruction were repeated for all saturation thresholds (40–90% of peak saturated peak) and arterial delay times, i.e., t_a_ (0–5 s) in Fig. 1. AIF curve candidates that failed to adhere to boundary constraints were discarded, and the one with the lowest cost function value was selected as the corrected AIF (Fig. 1). The resultant arterial (best fit) ${C}_{AIF}\left(t\right)$ were then used to calculate the perfusion parameters by the Fermi deconvolution approach [4].

### Patients

This study was approved by the Human Study Committee at our institution, and all patients gave written informed consent prior to participation. The study was also strictly compliant with the Health Insurance Portability and Accountability Act. Sixteen consecutive patients with a reversible myocardial perfusion defect visible on conventional single-photon emission computed tomography (SPECT) myocardial perfusion imaging (SPECT-MPI) were prospectively recruited (Table 1). The time difference between SPECT-MPI and PET-MRI examinations were within 10 days. The sixteen patients consisted of 7 men and 9 women (mean age, 58 years ± 12; range, 39–81 years). The inclusion criteria were reversible perfusion abnormalities at rest and regadenoson stress single-isotope SPECT-MPI perfusion defects in at least two contiguous myocardial segments. The exclusion criterion included: (a) subjects who suffered an intervening clinical event such as worsening angina pectoris or myocardial infarction or who underwent a myocardial revascularization procedure; (b) typical contraindications to MR imaging (pacemaker, brain aneurysm clips, shrapnel, etc.); (c) renal insufficiency (GFR < 60 mL/min/1.73m^2^) or other contraindication to gadolinium-based MR contrast agent; and (d) second or third degree atrioventricular (AV) block, active asthma, seizures, current hypotension (< 100/60), hypertension (> 160/90), pregnancy, breast feeding, the use of caffeine, nicotine or over the counter cold medicines within 12 h of the cardiac PET-MRI examination, and the use of dipyridamole within 48 h of the cardiac PET-MRI examination.

**Table 1:** Patient characteristics

	Patients (n = 16)
Age (years, ± SD)	58 ± 12
Male (%)	7 (44)
BMI (kg/m^2^, ± SD)	32.3 ± 6.0
eGFR (mL/min/m^2^, ± SD)	81 ± 18
Hypertension (%)	12 (75)
Diabetes (%)	4 (25)
Mean SPECT ejection fraction	57 ± 13%

### Cardiac PET-MRI protocol

All patients had an overnight fast of 6 h or more, except for water intake. The scanner was an integrated 3T PET-MRI system (Biograph mMR, Siemens Healthineers, Erlangen, Germany). Each patient underwent both rest and regadenoson-induced hyperemia stress perfusion imaging studies. The order of resting and hyperemia was randomly selected with a time difference of approximately 60 min.

Prior to perfusion imaging, scout images were first obtained, followed by attenuation correction scans using a two-point Dixon MRI method. Simultaneous acquisition of PET ^13^N-ammonia and gadolinium enhanced myocardial perfusion imaging were performed using the following methods. For simultaneous rest PET-MRI imaging, 10 mCi of ^13^N-ammonia and 0.075 mmol/kg of gadobenate dimeglumine (Multihance, Bracco Diagnostic, Monroe Township, New Jersey) at a rate of 5 mL/s followed by a 15 mL normal saline flush were administered into an antecubital vein. Simultaneously, a 10-min PET list-mode acquisition was initiated upon ^13^N-ammonia injection (using a second intravenous (IV) cannula). A saturation recovery turboFLASH CMR sequence was used to dynamically acquire 70 ECG triggered MRI images (per slice) for 3 short axis slices from the base to apical myocardium using the following parameters: TR/TE = 1.52/0.98 ms, FOV = 270 × 360 mm, matrix size of 144 × 192, sampling rate = 124 ms per image, isotropic pixel = 1.87 × 1.87 mm^2^, slice thickness = 6 mm, with parallel imaging acceleration factor of 2, and non-selective saturation recovery preparation pulse with TI = 100 ms, depending on the R-R interval. It is noted that a raw data filtering in the MRI system was used to reduce truncation artifacts. Stress imaging was performed using the same parameters as those listed above for the rest perfusion with the exception that regadenoson was administered (10–20 s) prior to the simultaneous IV injections of ^13^N-ammonia (10 mCi) and Multihance (0.075 mmol/kg). Regadenoson (400 µg) was administered in a single IV bolus (< 10 s) via an antecubital cannula, followed by 5 mL saline flush. Dynamic PET images were reconstructed with three-dimensional ordered-subset estimation-maximization (3D-OSEM) with 3 iterations, 21 subsets, with a post-reconstruction filter of 5 mm using a dynamic frames definition of 12 × 10 s, 4 × 30 s and 4 × 60 s using the clinical two-point Dixon attenuation correction.

### Image analysis

A standard American Heart Association (AHA) 16 segments approach was used for analysis on the same 3 slices from PET and MRI [13]. Absolute MBF of each segment from PET images was generated using QPET software package (Cedars-Sinai, Los Angeles, California, USA), based on the slice locations of CMR images. The Left Ventricle (LV) contour was automatically segmented from the sum dynamic images skipping the first 2 min and the LV input function VOI was automatically placed in the middle of the valve plane. The LV input function VOI was then manually adjusted to minimize spill over from surrounding myocardium and frame to frame motion correction was manually applied. MBF was computed using the standard 3 parameters, 2-compartment model with MBF, k_1_ (conversion of freely diffusible ^13^N-NH_3_ into metabolically bound ^13^N-glutamine, k_2_ (clearance) and blood spill-over correction as described in the literature [14, 15].

Two reviewers (JZ and ME with over 20 and 5 years of CMR experience, respectively) performed following independent image analysis. Dynamic CMR images were first manually registered to minimize respiratory motion. CMR perfusion images were then analyzed using an in-house software to quantify MBF in a segment-wise approach with Fermi deconvolution [4, 16]. The two reviewers, blinded to PET results and clinical history, manually drew the epi- and endo-myocardial contours at each image slice. The software automatically segmented the myocardial region into 6 or 4 segments per slice. An additional region-of-interest (ROI) was drawn in the LV pool to obtain the AIF. The average intensities of these myocardial segments and LV blood ROI were used to create signal-time curves to calculate MBF. The saturated and saturation-corrected AIFs were used to calculate MBFs as uncorrected MRI MBF and corrected MRI MBF, respectively. The inter-reviewer variability was assessed by the intra-class correlation for all MBF data at segment and slice basis.

Myocardial flow reserve (MFR) was quantified by the ratio of stress MBF to rest MBF in both PET and MRI. Additionally, pixel-wise MBF maps were also calculated from MR images, but only for visualization purposes. Care was taken to ensure the congruence of the MRI segments with the PET segments by an experienced observer (RL) prior to any analysis.

In addition to the quantitative comparison in MBF between CMR and PET, we also compared the area under the curve (AUC) of the AIF curves between PET and CMR acquisitions. To this end, the 10-min PET images at the basal slice location were resampled for the first 70 s with a temporal resolution (~ 1 s) that approximately matched with the temporal resolution of CMR. To ensure a fair comparison, AUC ratios (normalized to stress AUC curves) were calculated for PET and CMR AIF curves between two points (${i}_{1},{i}_{2}$) on the AIF curve used for CMR MBF quantification.

### Statistical analysis

Analyses were performed to assess differences between uncorrected MRI and corrected MRI MBF and PET MBF at rest and stress, as well as the difference in MFR between MRI and PET. Data was expressed as mean ± standard deviation. The accuracy of the CMR method was given by the correlation analysis through the Pearson’s correlation coefficient (r). Bland–Altman plots were obtained to evaluate the agreement between CMR and PET. These PET-CMR differences were also evaluated at different myocardial locations (basal, mid, apical locations). All statistical analyses were performed with JMP Pro Statistical Software (SAS Institute, Inc., Cary, NC), and MedCalc Statistics for Biomedical Research (MedCalc Software, Mariakerke, Belgium). A *p-*value < 0.05 was considered statistically significant.

## Results

**Fig. 2:**
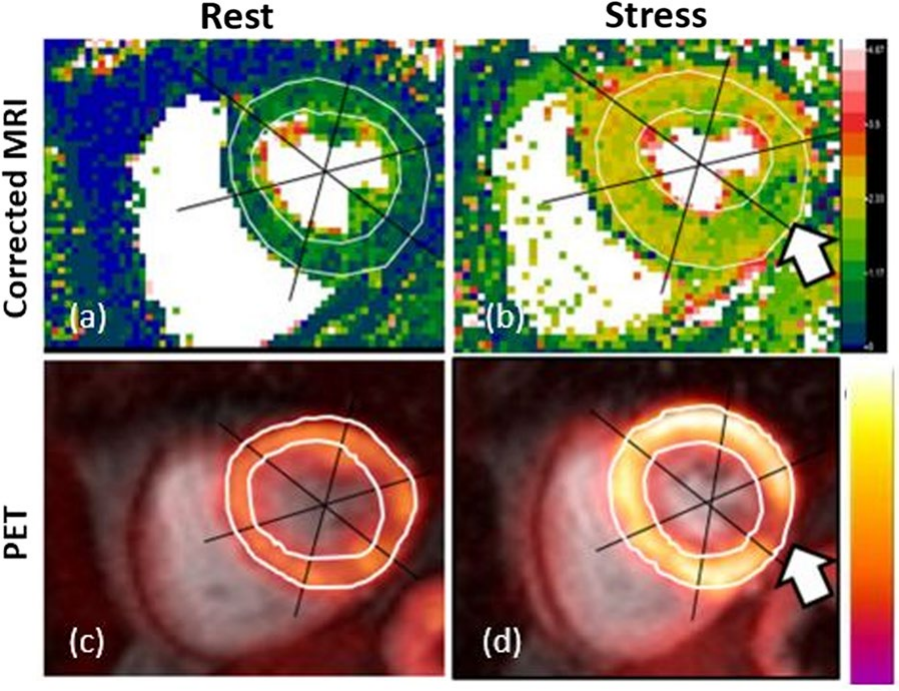
MBF maps (mid-level) at rest (**a**, **c**) and stress (**b**, **d**) derived from PET and CMR after AIF correction (voxel-wise). Ischemic regions (mid-inferolateral) in myocardium of a subject were identified by both PET and CMR MBF at stress (**b**) and (**d**) where CMR MBF maps were superior to PET MBF maps regarding with better spatial resolution. The color bar scales for both CMR and PET MBF are 0 – 4.5 ml/min/g

PET and CMR scans were successfully performed at rest and stress. The average time difference between rest and stress was 62 ± 7 min. The image quality was sufficient for contour detection to carry out manual image registration and myocardial segmentation in all slice locations. Figure 2 demonstrates examples of CMR MBF maps and corresponding PET images. While stress CMR MBF map showed the same ischemic region (mid-inferolateral) in myocardium as PET MBF map, it was evident that the stress CMR MBF map clearly delineates the ischemic territory with a higher spatial resolution.

### MBF

**Fig. 3:**
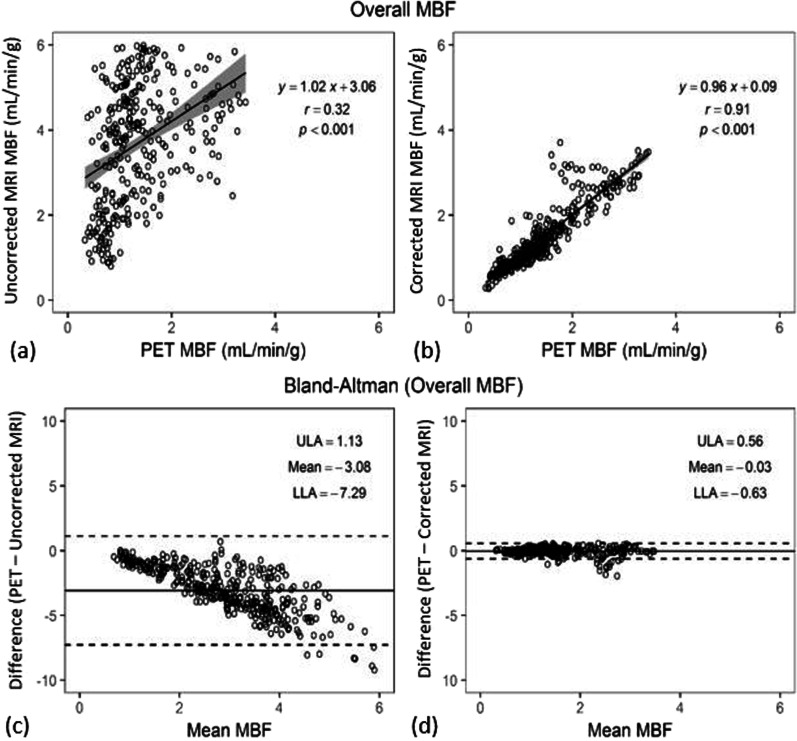
Linear regression (**a**, **b**) and Bland–Altman plots (**c**, **d**) of all MBF values of 16 patients showing the linear fit (with 95% CIs displayed as gray bands) and the agreement between PET and CMR before and after AIF corrections. Note that the MBF values derived after AIF corrections (corrected MRI) lie more uniform and closer to the horizontal axis (− 0.03) than in the Bland–Altman plot (bottom left) for uncorrected MRI. *r* = Pearson’s correlation. The *p* values are obtained with paired t test compared with PET MBFs. LLA and ULA represent Bland–Altman’s lower and upper limits of agreement, respectively

**Fig. 4:**
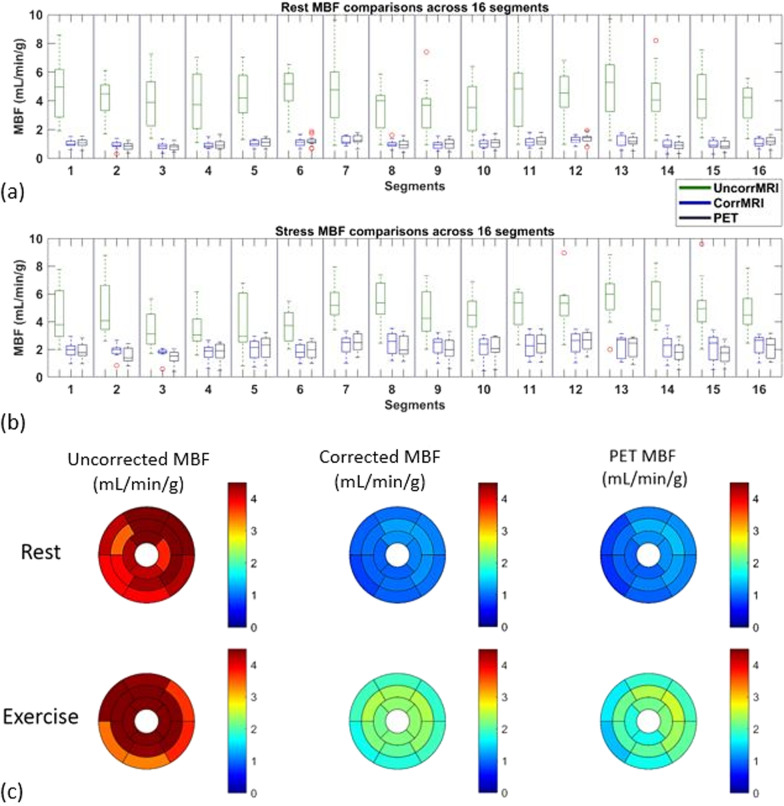
Boxplots show rest (**a**) and stress (**b**) MBF distribution and variability for all the myocardial segments before (uncorrected MRI; green) and after (corrected MRI; blue) AIF corrections, with PET MBF (black) as reference. Bullseyes plots (**c**) correspond to the MBF data shown in the boxplots for all the studied segments. The bullseyes plots of MBF by CMR after AIF correction approximately match those obtained from PET data. The color bar scale is 0–4.5 ml/min/g

The inter-class correlation was 0.82 (95% CI 0.78–0.86) and 0.9 (95% CI 0.83–0.94) for corrected MBF on a segment and slice basis, respectively. Overall, uncorrected MRI resulted in a larger MBFs (overestimation for 135–312%) but corrected MRI showed only slight overestimation of MBF (0–6%) when comparing to PET MBFs (Table 1). This is true for both resting MBF as well as stress MBF. Correlation analysis for MBF between CMR and PET demonstrated that MBF data from corrected MRI were uniformly distributed along the regression trend line with a strong correlation (*r* = 0.91, slope = 0.96, *P* < 0.001) (Fig. 3), while MBF data from uncorrected MRI dispersed largely with a weak correlation (*r* = 0.32, slope = 1.02, *P* < 0.001) (Table 2). Results of Bland–Altman analysis revealed concordance of segmental MBF measurements between corrected MRI and PET with narrow limits of agreement (− 0.63 to 0.56) and a mean difference (bias) of − 0.03 ± 0.30. The accuracy and agreement of resting and stress MBF values relative to PET MBF values are presented in Table 3, as well as in Table 4 at different myocardial slice locations. Figure 4 shows box plots of MBF values (uncorrected MRI, corrected MRI, and PET, from left to right in each segment column) at rest and stress, as well as Bull’s eye displays of these segmented data.

**Table 2:** Mean CMR MBFs calculated from uncorrected and corrected MRI, in comparison with PET

	Resting MBF	Stress MBF	Overall MBF*
Uncorrected MRI (± SD)	4.37 ± 2.49 (p < 0.001)	4.71 ± 1.82 (p < 0.001)	4.50 ± 2.26 (p < 0.001)
Corrected MRI (± SD)	1.04 ± 0.30 (p = 0.05)	2.12 ± 0.76 (p < 0.001)	1.45 ± 0.74 (p = 0.03)
PET (± SD)	1.06 ± 0.34	2.00 ± 0.74	1.42 ± 0.70

The p values indicate the comparisons in MBF between CMR and PET. * Overall MBF is the mean value of all segmented resting and stress MBF in all 16 patients

**Table 3:** Linear regression and agreement (Bland–Altman analysis) for rest and stress MBFs by MRI, compared to PET measurements

	Resting MBF		Stress MBF	
	PET vs uncorrected MRI	PET vs corrected MRI	PET vs uncorrected MRI	PET vs corrected MRI
Linear regression				
Slope	3.70	0.77	0.73	0.84
Intercept	0.44	0.23	3.26	0.43
Correlation coefficient (r)	0.51	0.89	0.29	0.83
Agreement				
Bias (± SD)	− 3.31 ± 2.33	0.02 ± 0.16	− 2.71 ± 1.75	− 0.12 ± 0.44
LLA	− 7.88	− 0.29	− 6.13	− 0.98
ULA	1.26	0.33	0.71	0.73

**Table 4:** Rest and stress MBFs derived from different myocardial locations

	Resting MBF			Stress MBF		
	Uncorrected MRI	Corrected MRI	PET	Uncorrected MRI	Corrected MRI	PET
Basal						
Mean ± SD	4.31 ± 1.76	0.98 ± 0.26	0.99 ± 0.31	3.95 ± 1.79	1.86 ± 0.57	1.77 ± 0.66
*P* value (vs PET)	< 0.001	0.656		< 0.001	0.034	
95% CI	− 3.66, − 2.99	− 0.03, 0.04		− 2.66, − 1.71	− 0.18, − 0.01	
Mid						
Mean ± SD	4.46 ± 3.37	1.10 ± 0.30	1.16 ± 0.36	5.01 ± 1.62	2.29 ± 0.79	2.26 ± 0.76
*P* value (vs PET)	< 0.001	0.004		< 0.001	0.521	
95% CI	− 3.98, − 2.64	0.02, 0.09		− 3.17, − 2.33	− 0.15, 0.08	
Apical						
Mean ± SD	4.33 ± 1.95	1.05 ± 0.34	1.05 ± 0.34	5.29 ± 1.82	2.23 ± 0.83	1.95 ± 0.74
*P* value (vs PET)	< 0.001	0.628		< 0.001	0.002	
95% CI	− 3.74, − 2.82	− 0.04, 0.02		− 3.92, − 2.76	− 0.46, − 0.11

**Fig. 5:**
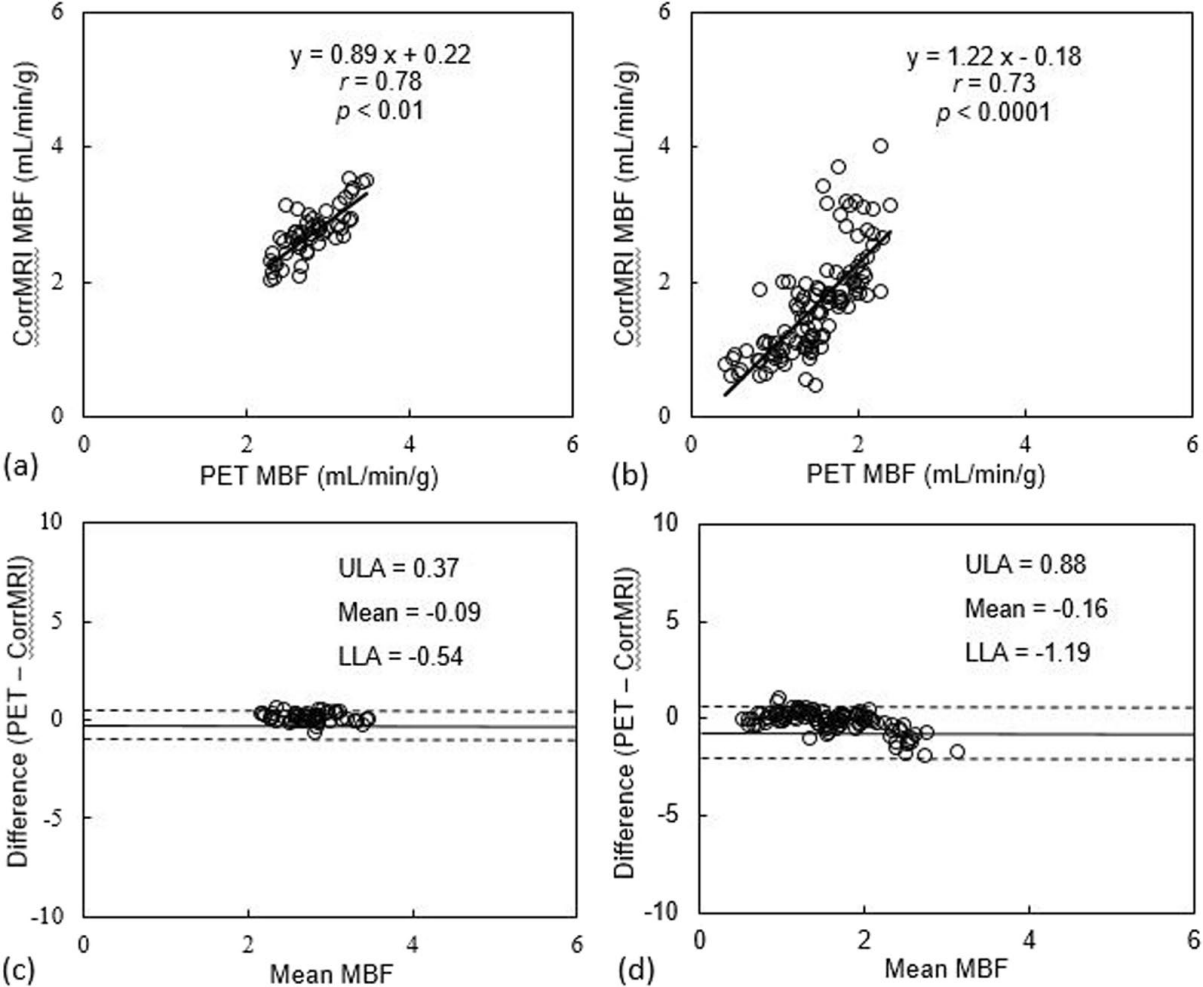
Linear regression (**a**, **b**) and Bland–Altman plots (**c**, **d**) of MBF values showing the linear fit and the agreement between PET and corrected MRI in non-ischemic (**a**, **c**) and ischemic segments (**b**, **d**). *r* = Pearson’s correlation. The *p* values are obtained with paired t test compared with PET MBFs. LLA and ULA represent Bland–Altman’s lower and upper limits of agreement, respectively

Based on the cut-off value of stress MBF of 2.3 mL/min/g for differentiation of ischemic from non-ischemic segments [17, 18], segmented stress MBF data was compared between corrected MRI and PET in ischemic and non-ischemia segments. There were strong correlations for both segments (*r* > 0.7), although corrected MRI slightly overestimated MBF in the ischemic segments (Fig. 5). The mean stress MBF values by corrected MRI (mL/min/g) were 2.72 ± 0.37 in non-ischemia segments and 1.66 ± 0.77 in ischemic segments. The corresponding MBF (mL/min/g) by PET were 2.80 ± 0.32 and 1.51 ± 0.45, respectively.

### Myocardial flow reserve

Compared with mean MFRs derived from PET (1.88 ± 0.67), the MFR from corrected MRI resulted in a slightly larger value (1.95 ± 0.66; *P* < 0.05), while the MFR from uncorrected MRI was considerably lower (1.44 ± 1.64; *P* < 0.001). There was a strong linear relationship between PET MFRs and corrected MRI MFRs (*r* = 0.82, *P* < 0.001) (Table 4). The agreement in MFRs between PET and corrected MRI demonstrated that the bias was relatively small (− 0.06 ± 0.40) and consistent across the entire range of mean MFR.

### AUC comparisons

In Table 5, linear regression analysis of AUC ratios showed statistically significant correlation between PET and corrected MRI (*r* = 0.84, *P* = 0.001). In contrast, the correlation coefficient for uncorrected MRI was 0.73 (*P* = 0.01). From the Bland–Altman analysis, the bias of MRI AUC was reduced from − 0.52 (uncorrected MRI) to 0.16 (corrected MRI).

**Table 5:** Comparisons of MFRs derived from uncorrected MRI, corrected MRI, and PET

	MFR		AUC ratio	
	PET-uncorrected MRI	PET-corrected MRI	PET-uncorrected MRI	PET-corrected MRI
Accuracy				
*p* value	< 0.001	< 0.001	0.011	0.001
Slope	1.41	0.81	0.45	1.06
Intercept	− 1.21	0.42	0.68	0.43
Correlation coefficient	0.57	0.82	0.73	0.84
Agreement				
Bias (± SD)	0.44 ± 1.37	− 0.06 ± 0.40	− 0.52	0.16
LLA	− 2.24	− 0.85	− 1.60	− 0.96
ULA	3.12	0.72	1.28	0.56

## Discussion

In this project, we have developed a post-processing method to retrospectively correct the saturated AIF in CMR perfusion studies when a high dose of contrast media is needed for better visualization of normal and ischemic myocardium. The corrected AIF using our simplified post-processing method can be used directly for the quantification of MBF using deconvolution methods (herein, we selected the Fermi method). The accuracy of this MBF quantification was validated by ^13^N-ammonia PET images that were simultaneously acquired with the CMR perfusion imaging. Three quantitative endpoints were obtained, MBF, MFR, and AUC ratios of AIF curves, from both CMR and PET analysis, in whole-hearts and from different segments (16 segments were adopted in this study). These endpoints in whole hearts between corrected CMR and PET reached mean differences (bias) of only − 0.03 (ml/min/100 g) for the absolute MBF, − 0.06 for MFR, and 0.16 for the AUC ratio. To our knowledge, this is the first time that a retrospective CMR AIF correction method has been validated against simultaneously acquired cardiac PET perfusion measurements.

While major approaches to obtain correct AIF are to prospectively acquire perfusion data with dual-bolus injections of contrast agents [5, 6] or with the use of dual-sequences [19], the proposed method allows direct correction of AIF from existing clinical perfusion data for the estimation of MBF and/or simplifies the CMR perfusion data acquisition without additional test bolus procedure or special pulse sequences. This work was built upon a method previously proposed in the liver [11] with two input functions (from hepatic artery and portal vein), but with only one input function obtained from LV blood pool. Unlike a previous model-based estimation of AIF in which multiple gamma-variate functions were included to represent the first and/or second pass of the contrast agent [20, 21], the current method accounts for only one gamma-variate function as the form of the first-pass AIF. The entire estimation was constrained by boundary and smoothness conditions listed in Eq. (6). After drawing the ROIs in blood pool and myocardial tissue, the computation of the AIF took approximately 1–2 s on a desktop computer (CPU, 4 GHz; RAM, 64 GB). Further studies could evaluate other analytical forms for calculation of AIF to improve the accuracy of MBF estimation, e.g., reduce the bias of MBF and MFR estimations.

Another noticeable advantage of this AIF correction method is that it is theoretically independent of the MRI system used, and the pulse sequence settings, as long as sampling rate is sufficiently high. Recently, an artificial intelligence-based AIF correction method was reported to estimate stress MBF by training data from perfusion imaging with the dual-bolus approach [22]. This fully automatic method demonstrated an excellent agreement in stress MBF between corrected AIF and dual-bolus methods. However, the method may need to be retained with data from different MRI system and/or pulse sequences. In addition, while we demonstrated this simplified AIF method with perfusion data using 0.075 mmol/kg gadobenate dimeglumine, it will also be interesting to explore this technique to correct AIF at different gadolinium dose levels.

One potential error is associated with the model assumption. We adopted a Fermi deconvolution method for MBF calculation, but the estimation of the AIF did not use any model for tissue kinetics. Using Fermi or other tracer kinetic models [23, 24], may introduce errors for the estimation of MBF, which is a subject of research in the future. However, we do expect these errors would be very small, given the excellent agreement between corrected MRI and PET measurements. Another potential error may arise when the stress perfusion measurement was performed first, followed by the resting perfusion measurement, due to the prolonged vasodilation effect of regadenoson. While this order of perfusion measurements is expected to introduce little error to the outcome since PET and MRI were acquired simultaneously, the residual vasodilatory effect on the accuracy of perfusion measurements will need more research with large patient numbers. Other potential errors associated with the MBF estimation include respiratory and cardiac motion. The former was mitigated by manual co-registration and the later was deemed minimal in our study. Nevertheless, residual uncorrected motion may still affect AIF and MBF estimation, particularly on tissue concentration time curves. The current automatic retrospective motion correction (MoCo) on perfusion images will greatly reduce such errors. Another potential source of error is the saturation effect of T2* decay which is more pronounced in AIF signals than in the myocardium, depending on magnetic field strength and specific contrast agents. When using gadobenate dimeglumine contrast agent with a relatively high T2 relaxivity [25], the AIF would be underestimated approximately 3.5% at a TE of 0.98 ms [26, 27], which may result in overestimation of MBF up to 10% without T2* correction [19]. At higher doses, this saturation effect needs to be considered when the non-saturated ranges are defined in the model. Other factors affecting the accuracy of AIF include sampling rate of perfusion imaging signals, signal-to-noise ratio, and AIF sampling location [28]. For instance, slow sampling rate would not provide accurate information of non-saturated portion of an AIF, which will result in partially or entirely wrong AIF correction. The AIF was selected in the basal level of LV, which may influence the accuracy of MBF calculation. Systematical investigation into these factors will be necessary to fully optimize this AIF correction technique.

There are several additional limitations to current study. First, we assume a linear relation of contrast agent concentration with observed tissue signal, which may need correction prior to the estimation of MBF [29]. Second, no coil sensitivity was considered prior to AIF estimation. Instead, a ring shaped ROI was selected in myocardial tissue of the short-axis image. The surface coil sensitivity for the mean intensity of this ROI would be similar to the coil sensitivity in the middle of the ROI, i.e., the location of blood pool AIF. A more accurate approach would be to perform a coil profile correction of the original perfusion images before AIF estimation. Third, there are still some apparent differences in MBF between PET and MRI (Fig. 4), e.g., stress MBF in the basal and apical segments. It is unknown if these differences, approximately 5–14% in segmental MBF, could significantly affect clinical diagnosis. It would be more clinically relevant to correlate MBF findings with coronary artery stenosis detected by invasive angiography or fractional flow reserve. A larger study is warranted to explore this quantitative perfusion technique for the detection of myocardial ischemia against reference methods. Lastly, PET MBF measurements are subject to motion, noise, and modeling errors. MBF measurements using invasive microsphere technique would be the true gold standard. Animal studies would be necessary to thoroughly validate the corrected MRI approach [30].

## Conclusion

In conclusion, this work presents a simplified but accurate method to estimate the true AIF based on saturated AIF signals in the first-pass perfusion imaging of myocardial tissue. The validation work through simultaneously acquired PET ^13^N-ammonia perfusion demonstrates strong agreement in perfusion and perfusion reserve. This post-processing technique has the potential to allow easy transformation of clinical myocardial perfusion MRI data into quantitative measurements.

## Data Availability

The data will be available from the corresponding author on reasonable request.

## Abbreviations

MBF: Myocardial blood flow
CMR: Cardiovascular magnetic resonance
PET: Positron emission tomography
LV: Left ventricle
AIF: Myocardial arterial input function
ROI: Region-of-interest
AUC: Area-under-curve
MFR: Myocardial flow reserve
